# Elevated plasma angiotensin converting enzyme 2 activity is an independent predictor of major adverse cardiac events in patients with obstructive coronary artery disease

**DOI:** 10.1371/journal.pone.0198144

**Published:** 2018-06-13

**Authors:** Jay Ramchand, Sheila K. Patel, Piyush M. Srivastava, Omar Farouque, Louise M. Burrell

**Affiliations:** 1 Department of Medicine, The University of Melbourne, Austin Health, Heidelberg, Victoria, Australia; 2 Department of Cardiology, Austin Health, Heidelberg, Victoria, Australia; The University of Tokyo, JAPAN

## Abstract

**Background:**

Angiotensin converting enzyme 2 (ACE2) is an endogenous regulator of the renin angiotensin system. Increased circulating ACE2 predicts adverse outcomes in patients with heart failure (HF), but it is unknown if elevated plasma ACE2 activity predicts major adverse cardiovascular events (MACE) in patients with obstructive coronary artery disease (CAD).

**Methods:**

We prospectively recruited patients with obstructive CAD (defined as ≥50% stenosis of the left main coronary artery and/or ≥70% stenosis in ≥ 1 other major epicardial vessel on invasive coronary angiography) and measured plasma ACE2 activity. Patients were followed up to determine if circulating ACE2 activity levels predicted the primary endpoint of MACE (cardiovascular mortality, HF or myocardial infarction).

**Results:**

We recruited 79 patients with obstructive coronary artery disease. The median (IQR) plasma ACE2 activity was 29.3 pmol/ml/min [21.2–41.2]. Over a median follow up of 10.5 years [9.6–10.8years], MACE occurred in 46% of patients (36 events). On Kaplan-Meier analysis, above-median plasma ACE2 activity was associated with MACE (log-rank test, p = 0.035) and HF hospitalisation (p = 0.01). After Cox multivariable adjustment, log ACE2 activity remained an independent predictor of MACE (hazard ratio (HR) 2.4, 95% confidence interval (CI) 1.24–4.72, p = 0.009) and HF hospitalisation (HR: 4.03, 95% CI: 1.42–11.5, p = 0.009).

**Conclusions:**

Plasma ACE2 activity independently increased the hazard of adverse long-term cardiovascular outcomes in patients with obstructive CAD.

## Introduction

Cardiovascular (CV) disease is a major cause of morbidity and mortality,[1] and is associated with activation of the renin-angiotensin system (RAS). Within the RAS, angiotensin converting enzyme (ACE) converts angiotensin (Ang) I to the vasoconstrictor and pro-atherosclerotic peptide Ang II,[2] whilst ACE2 is an endogenous inhibitor of the RAS through its major action to degrade Ang II.[3] ACE2 is highly expressed in the heart and blood vessels[4] and is cleaved from the cell surface to release the catalytically active ectodomain[5] into the circulation through the action of tumour necrosis factor alpha converting enzyme (TACE).[6] In human myocardium, ACE2 is localized to the endothelium of the microcirculation,[7] and is also present in the media of atherosclerotic radial arteries.[8] In healthy individuals, circulating ACE2 activity levels are low[9, 10] but increase in the presence of CV disease or risk factors including heart failure (HF),[11] atrial fibrillation (AF),[12] kidney disease[13, 14] and type 1 diabetes.[15] To date, there is limited information on the prognostic role of circulating ACE2 activity levels and the results are conflicting. For example, increased ACE2 activity predicted adverse CV outcomes in heart failure,[16] but not in patients after emergency orthopedic surgery[17] or with chronic kidney disease.[13, 14] These differences may reflect the patient population, the relative cardiovascular risk of the patient population or the length of follow up. The aim of this study was to investigate the utility of plasma ACE2 activity levels to predict CV events in a high-risk cohort of patients with angiographically proven obstructive CAD with more than 10 years of follow-up.

## Materials and methods

Consecutive patients aged >18 years were prospectively recruited between November 2004 and January 2006 after referral to a tertiary cardiovascular centre for a coronary angiogram to investigate suspected CAD. Only those with significant obstructive CAD were eligible for this study. Patients in cardiogenic shock, with a past history of congestive heart failure or with a left ventricular (LV) ejection fraction < 30% on angiography were excluded. Ethical approval was obtained from the Human Research Ethics Committee at Austin Health, Melbourne and the study complied with the Declaration of Helsinki. All patients gave informed written consent.

A standardised medical questionnaire was completed and verified with the hospital medical record. Blood pressure was measured and anthropometric measurements were taken. Obstructive CAD was defined as ≥50% stenosis of the left main coronary artery and/or ≥70% stenosis in ≥ 1 other major epicardial coronary artery by visual assessment on invasive coronary angiography.[18] Diabetes was diagnosed based on a documented history, treatment with glucose lowering therapy or if fasting blood glucose was >7 mmol/L. Hypertension was defined if previously diagnosed by a physician and/or current use of anti-hypertensive medication. Dyslipidaemia was defined if previously diagnosed by a physician and/or current use of lipid lowering agents. Cigarette smoking was defined as smoking within the preceding 12 months.

Fasting blood samples were collected at the time of admission for measurement of kidney function, lipids, and troponin. The Access AccuTnI assay (Beckman-Coulter, Chaska, MN, USA) was used to measure plasma troponin with the 99^th^ percentile of a healthy reference population of 0.04 μg/L. Levels of ≥ 0.04 μg/L (99^th^ percentile) were considered abnormal in this study.

For plasma ACE2 measurement, blood was collected within 48 hours of presentation into lithium heparin tubes, and plasma was obtained by centrifuging blood at 3000 rpm at 4°C for 10 minutes and stored at– 80°C until tested. Plasma ACE2 activity was measured within 2 years after all samples were collected. Samples were batched and ACE2 assays were run over a period of 2 days. The catalytic activity of ACE2 was measured using a validated, sensitive quenched fluorescent substrate-based assay as previously described.[9] Briefly, plasma (0.25 ml) was diluted into low-ionic-strength buffer (20 mmol/L Tris-HCl, pH 6.5) and added to 200 *μ*l ANXSepharose 4 Fast-Flow resin (Amersham Biosciences, GE Healthcare, Uppsala, Sweden) that removed a previously characterized endogenous inhibitor of ACE2 activity.[9] After binding and washing, the resulting eluate was assayed for ACE2 catalytic activity. Duplicate samples were incubated with the ACE2-specific quenched fluorescent substrate, with or without 100 mM ethylenediaminetetraacetic acid. The rate of substrate cleavage was determined by comparison to a standard curve of the free fluorophore, 4-amino-methoxycoumarin (MCA; Sigma, MO, USA) and expressed as *ρ*mole of substrate cleaved/mL of plasma/min. The intra-assay and inter-assay coefficient of variation was 5.6 and 11.8% respectively.

The primary endpoint was a composite of major adverse cardiac events (MACE) defined as CV death, hospitalisation for HF or myocardial infarction (MI). The secondary endpoint was HF hospitalisation. Endpoints were described according to the 2014 American College of Cardiology/ American Heart Association definitions for CV endpoints in clinical trials.[19] CV death was defined as death due to sudden cardiac death, HF, acute MI, cerebrovascular accident, CV haemorrhage, CV procedures or other CV causes, that is death not included in the previous categories but with a specific, known CV cause such as pulmonary embolism.[19] Hospitalisation for HF was defined as an event where the patient is admitted to the hospital with a primary diagnosis of HF where the length of stay is at least 24 hours, where the patient exhibits new or worsening symptoms of heart failure on presentation, has objective evidence of new or worsening heart failure and receives intensification of treatment specifically for heart failure.[19] Myocardial infarction was defined as the clinical diagnosis of ST elevation or non-ST elevation myocardial infarction according to established criteria.[20, 21]

Clinical outcomes were collected by an experienced blinded investigator via medical records review and by contacting each patient and/or the nominated general practitioner for additional information.

Statistical analysis was performed using STATA, version 14.2 (Statacorp., College Station, TX, USA). Normally distributed continuous variables are expressed as mean ± standard deviation and non-normally distributed data (Plasma ACE2 activity, triglycerides, troponin and glomerular filtration rate) are expressed as the median and inter-quartile range (IQR). Student *t* test or the Mann Whitney *U* test (for non-normally distributed data) was used to assess differences in continuous variables between patients with above and below median ACE2 activity. Categorical variables are expressed as counts and percentages and compared using Fisher’s exact or chi-square tests. Multiple regression analysis was used to identify variables that may independently influence plasma ACE2 activity. Plasma ACE2 activity, troponin levels and glomerular filtration rate were natural-logarithm transformed for analysis because of their skewed distribution. This rendered a more normal distribution by visual inspection of the distribution of the variables and Q-Q plots.

Cumulative incidence of MACE was estimated by the Kaplan Meier method and the log-rank test used to evaluate differences between patients with below and above median plasma ACE2 activity. When multiple end-points occurred during follow-up, the time to the first event was considered for analysis of MACE. Cox proportional hazard modelling was used to estimate the adjusted hazard ratio (HR) and 95% confidence interval (CI) for MACE. Significant variables (p < 0.1) from univariate analysis were entered into the final multivariate model to identify independent predictors of MACE. Conventional prognostic variables were used including age, history of diabetes, log troponin and treatment with statin, beta-blocker, ACE inhibitor or angiotensin receptor blocker in addition to log ACE2. Two-tailed p-values < 0.05 were considered significant.

## Results

We recruited 79 patients with angiographically proven obstructive CAD. No patient was lost to follow up and the median follow-up was 10.6 years (IQR 9.6–10.9 years). The clinical and biochemical characteristics of the study population are presented in Table 1. The cohort comprised 65% males with a mean ± SD age of 66 ± 12 years and BMI of 27.4 ± 4.4 kg/m^2^. Patients were at significant CV risk with 69% having a smoking history, and a history of CAD in 66%, dyslipidaemia in 60%, hypertension in 82%, diabetes in 24% and AF in 11%. With regard to pharmacological therapy at the time of presentation, 59% were on angiotensin converting enzyme inhibitors (ACEi) or angiotensin receptor blockers (ARB), 58% on beta-blockers, 72% on statins and 100% on aspirin.

**Table 1:** Participant characteristics in total cohort and according to plasma ACE2 activity.

Variable	All patients	ACE2 < median (n = 39)	ACE2 > median (n = 40)	P-value
Age (years)	66 ± 12	67 ± 11	63 ± 13	0.27
Male gender [n (%)]	51 (65%)	18(46%)	33 (83%)	0.001
BMI (kg/m^2^)	27.4 ± 4.4	27.4 ± 5.2	27.3 ± 3.6	0.939
SBP (mmHg)	130 ± 17	131 ± 15	130 ± 19	0.746
DBP (mmHg)	70 ± 13	71 ± 13	68 ± 14	0.321
Presentation with ACS	51 (65%)	23 (59%)	28 (70%)	0.306
*Previous history* [n (%)]				
CAD	51 (66%)	24 (63%)	27 (69%)	0.573
Dyslipidemia	48 (60%)	23 (59%)	25 (63%)	0.748
Hypertension	64 (82%)	30 (77%)	34 (87%)	0.238
Diabetes	19 (24%)	11 (29%)	8 (20%)	0.357
Atrial fibrillation	9 (11%)	1 (3%)	8 (20%)	0.015
Smoking history	54 (69%)	24 (63%)	30 (75%)	0.257
*Medical treatment* [n (%)]				
ACEi/ARB	45 (59%)	24 (63%)	21 (55%)	0.484
Beta-blocker	44 (58%)	23 (61%)	21 (55%)	0.642
Statin	56 (72%)	27 (69%)	29 (74%)	0.615
Aspirin	79 (100%)	39 (100%)	40 (100%)	1
Serum biochemistry				
LDL (mmol/L)	2.5 ± 0.9	2.6 ± 1.1	2.5 ± 0.8	0.736
Triglycerides (mmol/L)	1.2 [0.9–1.9]	1 [0.8–1.9]	1.4 [0.9–2]	0.119
eGFR (ml/min/1.73m^2^)	70 [48–96]	76 [59–98]	69 [46–95]	0.675
Troponin I (μg/L)	0.6 [0–14.5]	0.54 [0–8.2]	2.3 [0–21.2]	0.211
LVEF < 50% [n (%)]	42 (58%)	18(53%)	24 (62%)	0.459
Culprit vessel [n (%)]				
Right coronary	27 (34)	15 (39)	12 (30)	0.428
Left anterior descending	23 (23)	13 (33)	10 (25)	0.415
Left circumflex	20 (25)	8 (21)	12 (30)	0.332
Left main	1 (1)	0	1 (3)	0.320
Inpatient revascularisation [n (%)]	61 (77)	31 (79)	30 (75)	0.635
MACE [n (%)]	36 (46%)	12 (31%)	24 (60%)	0.009

Values are mean ± standard deviation, n (%) or median [interquartile range].

ACS = acute coronary syndrome; BMI = body mass index; ACEi = angiotensin converting enzyme inhibitor; ARB = angiotensin receptor blocker; CAD = coronary artery disease; DBP = diastolic blood pressure; eGFR = estimated glomerular filtration rate; LVEF = left ventricular ejection fraction; LDL = low-density lipoprotein; MACE = major adverse cardiovascular events; SBP = systolic blood pressure

There was no difference in plasma ACE2 levels between patients with ST elevation acute coronary syndrome (ACS) [n = 24, median 31.7 pmol/ml/min (IQR 5.6–43.9)], non-ST elevation ACS [n = 27, median 29.1 pmol/ml/min (IQR 23.1–46.4)] or stable angina [n = 28, median 26.7 pmol/ml/min (IQR 18.7–39.8), p = 0.386] and so further analysis was conducted in the whole cohort.

The median ACE2 level in the whole cohort was 29.3 pmol/ml/min [IQR 21.2–41.2]. Patients were categorized according to plasma ACE2 activity above / below the median ACE2 level. Patients with above–median plasma ACE2 activity were more likely to be male and have AF (Table 1, both p < 0.05). Multiple regression analysis was performed to identify variables that influence plasma ACE2 activity. Male gender was the only independent predictor of higher ACE2 activity (p = 0.022). The prevalence of CAD and cardiac risk factors including dyslipidaemia, hypertension, diabetes and cigarette smoking were similar in the two groups, as was LVEF <50%, the use of pharmacological agents, low density lipoprotein cholesterol, triglycerides levels, kidney function and troponin level (all p > 0.05).

Over the follow-up period, there were 18 deaths, 19 myocardial infarcts and 16 hospitalisations with HF. The primary endpoint of MACE, a composite of CV mortality, HF hospitalisation or MI occurred in 36 patients (46%).

**Fig 1:**
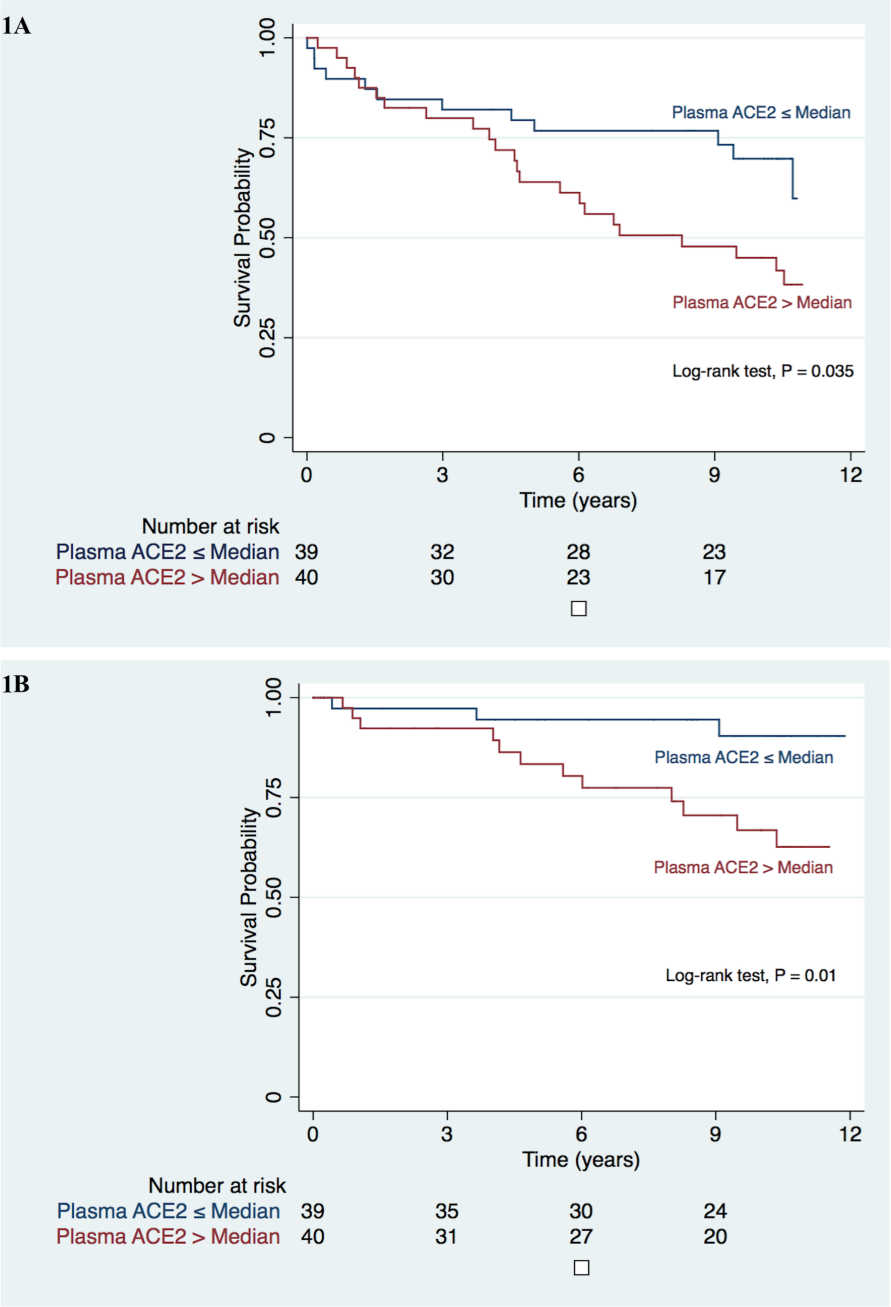
Clinical events at follow-up. Kaplan Meier Survival plot illustrating event free survival from major adverse cardiac events (A) and heart failure hospitalisation (B) in patients with coronary artery disease, stratified according to median plasma ACE2 level of 29.3 pmol/ml/min. ACE2 *=* angiotensin converting enzyme 2.

Above median levels of ACE2 (>29.3 pmol/ml/min) were significantly associated with a higher incidence of MACE (log-rank test, p = 0.035; Fig 1A) and HF hospitalisation (p = 0.01; Fig 1B) compared with those with below-median ACE2. There was no significant difference in the incidence of CV death (p = 0.195) or MI (p = 0.35). In a subgroup analysis including male patients only, there was no significant difference in the incidence of MACE according to median levels of ACE2 (p = 0.124).

Survival analysis using the Cox regression model indicated that age, history of atrial fibrillation, history of diabetes and log ACE2 were univariate predictors of the primary endpoint of MACE. On multivariable Cox regression analysis, Log ACE2 activity remained the only significant predictor of MACE (HR: 2.4; 95% CI: 1.24 to 4.72; p = 0.009) (Table 2).

**Table 2:** Cox regression analysis for MACE in patients with obstructive CAD.

Variable	Unadjusted HR (95% CI)	Unadjusted p Value	Adjusted HR (95% CI)	Adjusted p Value
Age	1.03 (1.0–1.06)	0.068	1.03(1–1.06)	0.129
Male	1.36 (0.65–2.83)	0.413		
Atrial fibrillation	2.26 (0.98–5.21)	0.053	1.49 (0.63–3.52)	0.364
Diabetes	1.92 (0.96–3.87)	0.067	1.88 (0.93–3.78)	0.078
ACEi/ARB	0.64 (0.32–1.26)	0.195		
ß-blocker use	0.57 (0.29–1.13)	0.107		
Statin use	0.65 (0.32–1.32)	0.237		
Log troponin	1.07 (0.97–1.18)	0.194		
Log ACE2	2.56 (1.31–5)	0.006	2.42 (1.24–4.72)	0.009

ACE2 = angiotensin converting enzyme 2; ACEi = angiotensin converting enzyme inhibitor; ARB = angiotensin receptor blocker; MACE = major adverse cardiovascular events

With regard to the secondary endpoint of HF, both log ACE2 (HR: 4.03; 95% CI: 1.42–11.5; p = 0.009) and age (HR: 1.06; 95% CI: 1.01–1.12, p = 0.026) were independent predictors of heart failure.

## Discussion

The major finding of the current study was that plasma ACE2 activity independently increased the hazard for adverse cardiovascular events in patients with significant obstructive CAD. In this study in high-risk patients followed for a median of 10.6 years, elevated ACE2 activity remained an independent predictor of CV mortality and morbidity even after comprehensive multivariable adjustment in a model that included prognostically meaningful variables. The median ACE2 level in patients with CAD was 29 pmol/ml/min which is higher than levels we previously reported in young healthy volunteers (4.44 ± 0.56 pmol/ml/min)[9] and in elderly patients (median 19.4 pmol/ml/min).[17] We excluded patients with known HF or severe LV systolic dysfunction as both are associated with increased circulating ACE2 levels.[11, 16] Consistent with results of other studies,[13–15, 22] plasma ACE2 activity was higher in male patients but we found no other independent predictors of plasma ACE2 activity levels.

There are conflicting findings regarding the prognostic value of circulating ACE2 levels likely reflecting the differences in follow-up period and risk of CV events across the study populations. In a cohort of patients with HF (n = 113), 23% had an adverse CV event (death, cardiac transplant, HF hospitalisation) over a 34 month follow up and circulating ACE2 levels remained an independent predictor after adjustment for reduced ejection fraction and increased N-terminal-pro brain natriuretic peptide.[16] In another cohort of patients with chronic kidney disease (CKD) without prior CV disease, circulating ACE2 activity was not an independent predictor of CV mortality or events over a follow-up period of 24 months.[13, 14] In concordance, our group reported no significant associations between elevated circulating ACE2 activity and adverse CV outcomes in patients with CKD stage III/ IV, haemodialysis patients or kidney transplant recipients.[13] We also found that in elderly patients undergoing emergency orthopaedic surgery, elevated ACE2 levels did not predict CV events after 12 months of follow-up (p = 0.051).[17] We ascribe the significant association between increased plasma ACE2 activity and adverse CV outcomes seen in the present study to a higher rate of CV outcomes observed in the study cohort and longer follow-up duration.

Severe lines of evidence suggest that plasma ACE2 activity may serve as a marker of atherosclerosis. In non-dialysis patients with CKD, circulating ACE2 activity was associated with silent atherosclerosis in carotid and peripheral vessels.[14] In patients with type 1 diabetes and a history of CAD, circulating ACE2 activity was increased.[15] The same pattern was observed in kidney transplant recipients with a history of CAD,[23] further supporting the association between raised circulating ACE2 activity and coronary atherosclerosis. In another study of patients with angiographically confirmed CAD, Ortiz-Perez et al. demonstrated elevated levels of circulating ACE2 at baseline (24-48h) in patients presenting with ST-elevation myocardial infarction compared to a control group of patients without known CAD.[24] It is not therefore clear from the Ortiz-Perez et al. study whether the increase in circulating ACE2 reflects acute cardiac injury or underlying atherosclerosis. Our study extends knowledge in this regard as we included only patients with angiographically proven obstructive coronary artery disease, both with and without an acute presentation. As there was no difference in ACE2 according to presentation, our results suggest that the increase in plasma ACE2 reflects underlying atherosclerosis rather than acute myocardial injury.

The importance of the RAS in the pathogenesis of atherosclerosis is well established and indeed targeted pharmacological inhibition of the classic RAS improves outcomes in atherosclerotic disease including CAD.[25] In experimental models of atherosclerosis, we and others reported that ACE2 is expressed in vascular endothelial cells, macrophages and smooth muscle cells within atherosclerotic plaques.[26, 27] We also reported that ACE2 was present in atherosclerotic blood vessels in patients with CAD undergoing coronary artery bypass surgery.[8] Experimental studies have shown that ACE2 overexpression promotes atherosclerotic plaque stability and attenuates atherosclerotic lesions[28, 29]. Activation of TACE results in increased ACE2 shedding from tissue into the circulation.[6] Shedding and hence loss of ACE2 from the tissue is mediated by angiotensin II and results in the pro-inflammatory effects of angiotensin II being unopposed.[6] Certainly in a rabbit model of atherosclerosis, gene silencing of TACE enhanced plaque stability and improved vascular remodelling,[30] possibly via reduced tissue ACE2 shedding. These findings reinforce the important counter-regulatory role of ACE2 in atherosclerosis and suggest that modulation of ACE2 could offer a future therapeutic option in patients with atherosclerotic disease.

The relationship between tissue and circulating levels of ACE2 is not yet understood. It has been postulated plasma ACE2 levels may parallel tissue ACE2 expression with a constant rate of shedding in normal physiology[16], although there are no studies that have concurrently measured both tissue and circulating ACE2 and TACE levels to address this hypothesis. Our findings raise the possibility that in human atherosclerosis, increased plasma ACE2 activity in those with adverse cardiovascular outcomes reflect a persistent albeit insufficient counter-regulatory process to shift the balance away from the deleterious effects of sustained Ang II activation. Genetic variation in and around the gene encoding ACE2 may account for differences in ACE2 expression or activity. Indeed, the location of the ACE2 gene within the X chromosome in an area where genes are known to escape X-inactivation may contribute to phenotypic differences between sexes and tissue-specific differences in X-inactivation.[31] Furthermore, the rs1978124 polymorphism in the *ACE2* gene has been associated with poorer outcomes in two separate CAD cohorts of Chinese Han[32] and European[33] ancestry but there are not yet studies that combine genetic approaches with measurement of plasma ACE2 activity.[34]

The study has a number of limitations including the relatively small sample size and the use of a conventional troponin assay, as a high sensitivity assay was not available at the time of patient recruitment. Furthermore, the finding of elevated plasma ACE2 activity and its association with adverse outcomes only suggest a possible relationship and does not determine cause or effect. However major strengths include the detailed angiographic assessment and the long term follow up.

In conclusion, our study demonstrates that elevated plasma ACE2 activity is an independent predictor of MACE in patients with obstructive CAD.

### Future

This study has identified ACE2 as a potential surrogate marker of CV outcomes, and possibly a target for therapeutic intervention. Whether targeting patients with increased plasma ACE2 levels for more intensive therapy would lead to improved outcomes has yet to be tested.
